# Does surgery for instability of the shoulder truly stabilize the glenohumeral joint? A prospective comparative cohort study

**DOI:** 10.1097/MD.0000000000004369

**Published:** 2016-08-07

**Authors:** Alexandre Lädermann, Patrick J. Denard, Jérôme Tirefort, Frank C. Kolo, Sylvain Chagué, Grégory Cunningham, Caecilia Charbonnier

**Affiliations:** a Division of Orthopaedics and Trauma Surgery, Clinique La Colline; b Faculty of Medicine, University of Geneva; c Division of Orthopaedics and Trauma Surgery, Department of Surgery, Geneva University Hospitals, Geneva, Switzerland; d Southern Oregon Orthopedics, Medford; e Department of Orthopaedics and Rehabilitation, Oregon Health & Science University, Portland, OR; f Rive Droite Radiology Center; g Artanim Foundation, Medical Research Department, Geneva, Switzerland.

**Keywords:** 3D simulation, apprehension, biomechanics, computer tomography, dislocation, glenohumeral stabilization, kinematics modeling, motion capture, subluxation, subtle or minor instability, unstable painful shoulder

## Abstract

Despite the fact that surgery is commonly used to treat glenohumeral instability, there is no evidence that such treatment effectively corrects glenohumeral translation. The purpose of this prospective clinical study was to analyze the effect of surgical stabilization on glenohumeral translation.

Glenohumeral translation was assessed in 11 patients preoperatively and 1 year postoperatively following surgical stabilization for anterior shoulder instability. Translation was measured using optical motion capture and computed tomography.

Preoperatively, anterior translation of the affected shoulder was bigger in comparison to the normal contralateral side. Differences were significant for flexion and abduction movements (*P* < 0.001). Postoperatively, no patients demonstrated apprehension and all functional scores were improved. Despite absence of apprehension, postoperative anterior translation for the surgically stabilized shoulders was not significantly different from the preoperative values.

While surgical treatment for anterior instability limits the chance of dislocation, it does not seem to restore glenohumeral translation during functional range of motion. Such persistent microinstability may explain residual pain, apprehension, inability to return to activity and even emergence of dislocation arthropathy that is seen in some patients. Further research is necessary to better understand the causes, effects, and treatment of residual microinstability following surgical stabilization of the shoulder.

## 1 Introduction

Glenohumeral dislocation affects 1.7% of the general population, making instability of the shoulder the most frequent of all joint instabilities.^[1]^ While surgical stabilization can dramatically reduce the risk of recurrent dislocation, many patients can remain symptomatic. Shoulder apprehension is defined as a fear of dislocation and/or resistance in patients with a history of anterior glenohumeral instability. After an open or arthroscopic stabilization, 3% to 51% of the patients will keep apprehension or will avoid shoulder movement because of fear of dislocation.^[2,3]^ Such symptoms can lead to decreased activity, prolonged absence from work and sports, and a general decrease in quality of life.^[4,5]^

**Figure 1:**
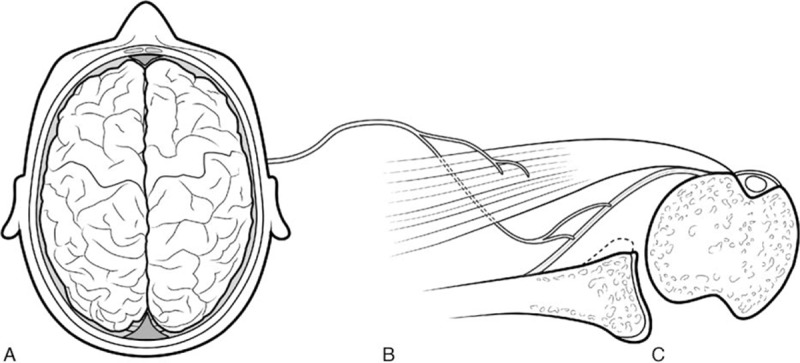
Apprehension could be related to (A) central nervous system sequelae, (B) peripheral neurological, muscular or capsular/ligamentous lesions consecutively to dislocation, or (C) mechanical instability as micromovements.

Currently, the source of persistent postoperative apprehension has not been well studied. Theoretically, such apprehension after glenohumeral stabilization could be related to central nervous system sequelae secondary to a traumatic dislocation event,^[6,7]^ peripheral neurological lesion consecutively to dislocation affecting proprioception,^[8]^ or actual mechanical instability of the glenohumeral joint (Fig. 1).^[9,10]^ Optical motion capture is a noninvasive technology that offers ability to measure glenohumeral translation^[11]^ and may help differentiate supratentorial apprehension from true mechanical instability.

The purpose of this study was to evaluate glenohumeral translation in patients suffering from anterior instability and analyze the effect of glenohumeral stabilization on this translation. The hypothesis was that surgical stabilization only partially corrects glenohumeral translation in unstable shoulders.

## 2 Methods

### 2.1 Patient selection

This was a prospective evaluation of a consecutive series of patients who underwent surgical stabilization for glenohumeral instability. Institutional review board approval was obtained before study beginning (AMG 12–18), and the subjects signed a written informed consent form before participation. All patients were evaluated by the primary author (AL). Inclusion criteria were a primary surgery, traumatic anterior glenohumeral instability, and age between 15 and 65 years. We excluded patients with incomplete documentation, follow-up of less than 12 months, history of bilateral instability, previous shoulder surgery, contraindication for computed tomography (CT), nontraumatic onset, and hyperlaxity. The latter was defined as more than 85° of external rotation with the elbow at the side.^[12]^

### 2.2 Operative technique

All operations were performed in a semibeach chair position under general anesthesia with a single-shot interscalene block or continuous catheter. Glenohumeral stabilization was performed with either an open or arthroscopic technique. Open Latarjet was performed as classically described with a subscapularis split and triple locking mechanism approach.^[13]^ The graft was intraarticular in every case, the capsule was systematically reattached to glenoid according to Favard modification,^[14]^ and a capsular shift was added. Arthroscopic Latarjet was carried out according to a modified Lafosse technique.^[15]^ In the latter treatment option, no reattachment of the capsule was performed. In both arthroscopic and open techniques, the patients were postoperatively protected with a sling for 10 days and were able to immediately start full active range of motion (ROM). Return to low-risk sports was allowed at 6 weeks, and high-risk (throwing and collision) sports at 3 months. The arthroscopic Bankart repair consisted of a mobilization of the anteroinferior capsule and the labrum with an arthroscopic elevator. The glenoid rim and neck were then prepared with a mechanical shaver device. Two double-loaded anchors were inserted at the 5- and 3-o’clock positions, and sutures were shuttled across the inferior glenohumeral ligament and labrum, starting at the inferior position and progressing in a superior direction. Postoperatively, the arm was protected in a sling for 4 weeks. Return to low-risk sports was allowed at 10 weeks, and high-risk (throwing and collision) sports at 4.5 months.

### 2.3 Study outcomes

The main outcomes of interest were pre- and postoperative ipsilateral glenohumeral translation, as well as contralateral glenohumeral translation. Furthermore, the prevalence of postoperative apprehension, recurrent dislocation or subluxation, and ROM in the normal and the unstable shoulder were evaluated in relation to the main outcomes of interest. The following baseline characteristics were assessed: age, gender, shoulder side, and limb dominance.

### 2.4 Clinical evaluation

Two orthopedic surgeons independent to the operating surgeon performed all physical examinations. Clinical examination included assessment of rotator cuff strength, shoulder ROM, and anterior apprehension (graded as positive or negative). Walch-Duplay^[16]^ and Rowe scores,^[6,17]^ the subjective shoulder value (SSV, a single-question test where the patient is asked to rate his overall shoulder function as a percentage of normal shoulder),^[18]^ and a visual analog scale (VAS) pain score graded from 0 point (no pain) to 10 points (maximal pain)^[19]^ were recorded.

### 2.5 Radiographic evaluation

All volunteers underwent a preoperative CT of bilateral shoulders and arms. The CT examinations were conducted with a LightSpeed VCT 64 rows system (General Electric Healthcare, Milwaukee, WI). Images were acquired at 0.63 mm slice thickness. Based on the CT images, patient-specific three-dimensional (3D) models of the shoulder bones (humerus, scapula, clavicle, and sternum) were reconstructed for each patient using Mimics software (Materialize NV, Leuven, Belgium).

### 2.6 Motion capture

All patients participated in motion capture sessions preoperatively and 1 year postoperatively. Kinematic data were recorded using a Vicon MX T-Series motion capture system (Vicon, Oxford Metrics, UK) consisting of 24 T40S cameras sampling at 120 Hz. The patients were equipped with a previously described shoulder markers protocol,^[11]^ which included 69 spherical retroreflective markers. The setup included 4 markers (Ø 14 mm) on the thorax (sternal notch, xyphoid process, C7 and T8 vertebra), 4 markers (Ø 6.5 mm) on the clavicle, 4 markers (Ø 14 mm) on the upper arm—2 placed on the lateral and medial epicondyles and 2 as far as possible from the deltoid—and 57 markers on the scapula (1× Ø 14 mm on the acromion and a 7 × 8 grid of Ø 6.5 mm). Finally, additional markers were distributed over the body (nondominant arm and legs) to provide a global visualization of motion.

During each session the patients were asked to perform the following motor tasks (3 trials each): internal–external rotation with 90° abduction and the elbow flexed 90°, internal–external rotation with the arm at the side, forward flexion of the arm from neutral to maximum flexion, and empty-can abduction from neutral to maximum abduction in the scapular plane. Both shoulders (normal and unstable) were measured during the first session, whereas only the surgically stabilized shoulder was assessed postoperatively. The same investigators (CC, SC) attached all markers and performed all measurements.

### 2.7 Kinematic analysis

**Figure 2:**
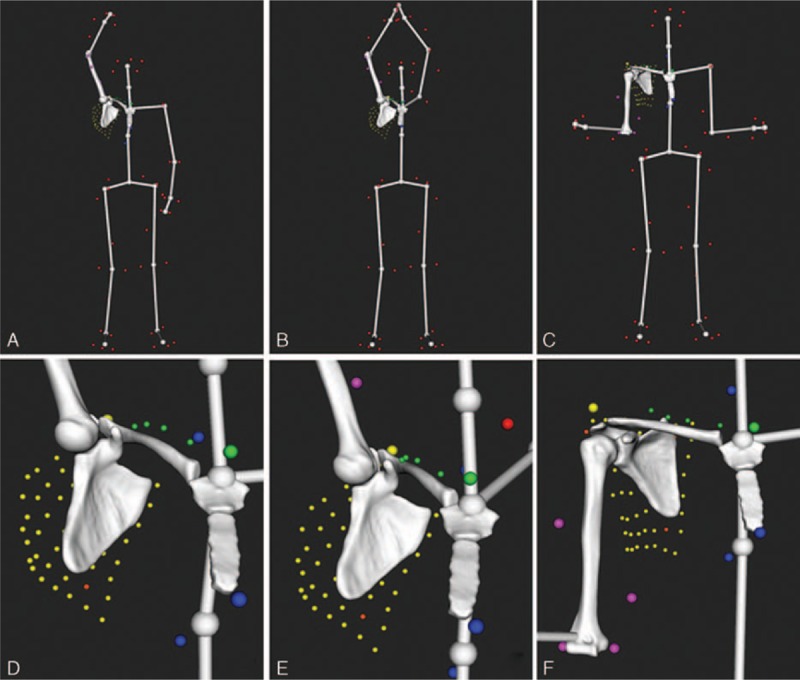
Examples of computed postures on a right shoulder showing the markers setup (small colored spheres) and a virtual skeleton used to better visualize the motion as a whole: (A) maximum flexion, (B) maximum abduction in the scapular plane, (C) maximum external rotation with elbow at side, (D), (E), and (F) show a zoom in the shoulder for each posture (A), (B), and (C), respectively.

Shoulder kinematics was computed from the recorded markers’ trajectories using a validated biomechanical model which accounted for skin motion artifact.^[11,20]^ The model was based on a patient-specific kinematic chain using the shoulder 3D models reconstructed from the CT data and a global optimization algorithm with loose constraints on joint translations (accuracy: translational error < 3 mm, rotational error < 4°). Figure 2 shows examples of computed postures.

Maximal glenohumeral ROM was quantified for flexion, abduction, internal and external rotation, and expressed in clinical terms.^[21]^ This was achieved by calculating the relative orientation between 2 local coordinate systems, 1 for the scapula and 1 for the humerus, based on the definitions suggested by the International Society of Biomechanics.^[22]^ The local systems were created using anatomical landmarks identified on the patient's bony 3D models. The glenohumeral joint center was calculated based on a sphere fitting method.^[23]^ To facilitate clinical comprehension and comparison, motion of the humerus with respect to the thorax was also calculated. This was obtained with the same method by using thorax and humerus coordinate systems.

**Figure 3:**
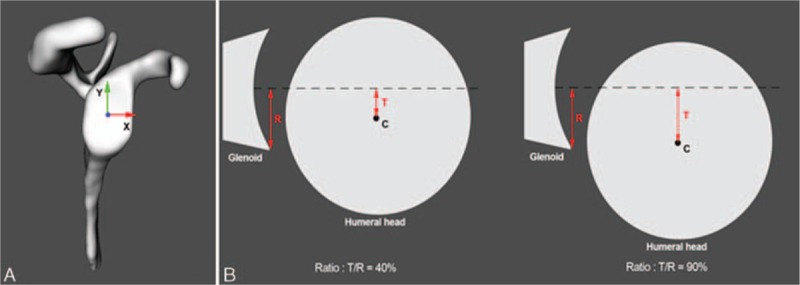
(A) Definition of the glenoid coordinate system used in this study. (B) Schematic representation of glenohumeral subluxation (C = center of the humeral head, R = radius of the width or height of the glenoid surface, T = translation of the humeral head center). Left: the ratio is 40%, there is no instability. Right: the ratio is >50%, instability is noted. Image reproduced from Lädermann et al^[24]^ with permission.

Glenohumeral translation, defined as anterior–posterior and superior–inferior motion of the humeral head center relative to the glenoid coordinate system,^[24]^ was assessed at maximal ROM during all tested movements. The coordinate system was determined by an anterior–posterior x-axis and a superior–inferior y-axis with an origin placed at the intersection of the anteroposterior and superoinferior aspects of the glenoid rim (Fig. 3A). Subluxation was defined as the ratio between the translation of the humeral head center and the radius of width (anteroposterior subluxation) or height (superoinferior subluxation) of the glenoid surface (Fig. 3B). Instability was defined as subluxation bigger than 50%.^[25]^

Glenohumeral ROM, humeral motion relative to the thorax, glenohumeral translation, and subluxation were computed for all patients and selected for statistical analysis.

### 2.8 Statistical analysis

Statistical analysis was performed using software package R, v3.1.2 Portable (Free Software Foundation, Inc., Vienna, Austria). Descriptive statistics was presented in terms of mean and standard deviation (SD). Shapiro–Wilk test was used to screen the outcomes for normal distribution. Paired samples *t* tests were applied to check for significant differences of the kinematic data between the normal and unstable shoulders, as well as between the pre- and postoperative pain scores. Level of significance was set at *P* < 0.05.

## 3 Results

**Figure 4:**
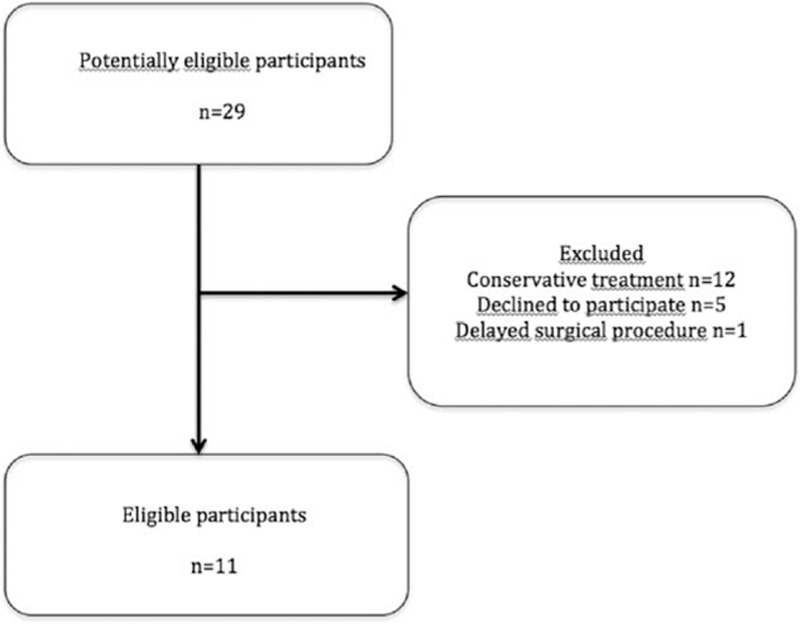
STARD flow diagram.

Between October 2014 and January 2015, 29 patients were admitted in our clinic with shoulder instability. Seventeen patients met the inclusion study criteria (Fig. 4). Five patients declined to participate and 1 postponed the surgical procedure. Therefore, 11 patients were included in the study (10 males and 1 female) with a mean age of 26.6 years (range, 17–44 years). Ten patients had right-sided glenohumeral instability and 1 patient was with left-sided glenohumeral instability. The dominant side was involved in 8 cases. Four patients were involved in competitive sports and 7 participated only in recreational activities. Preoperatively, the patients experienced a mean of 6.0 ± 5.7 (mean ± SD) dislocations. An open Latarjet technique was used to stabilize the shoulder in 9 cases, an arthroscopic Latarjet was used in 1 case, and an arthroscopic Bankart repair was performed in 1 case. Mean follow-up was 13 months (range, 12–14 months).

### 3.1 Functional outcome

No patients had recurrent dislocation during the study period or had a positive apprehension sign on the final follow-up. Compared to the preoperative values, the postoperative VAS pain score improved from 2.5 ± 2.1 to 0.1 ± 0.3 (*P* = 0.002), the SSV score from 50.5 ± 25.1 to 96.3 ± 5.0 (*P* < 0.001), and the Rowe score from 30.5 ± 14.9 to 99.5 ± 1.5 (*P* < 0.001). Pre- and postoperative ROM are detailed in Table 1. In general, preoperative ROM of the normal shoulder was greater than the unstable shoulder. Postoperative ROM of the surgically treated shoulders was less than the normal shoulder. This was significant for flexion, abduction, and external rotation with the arm at the side. Postoperative ROM was not statistically different from the preoperative ROM, except for the humerus motion relative to the thorax during flexion (*P* = 0.009) and internal rotation of the arm with 90° abduction and the elbow flexed 90° (*P* = 0.004).

**Table 1:**
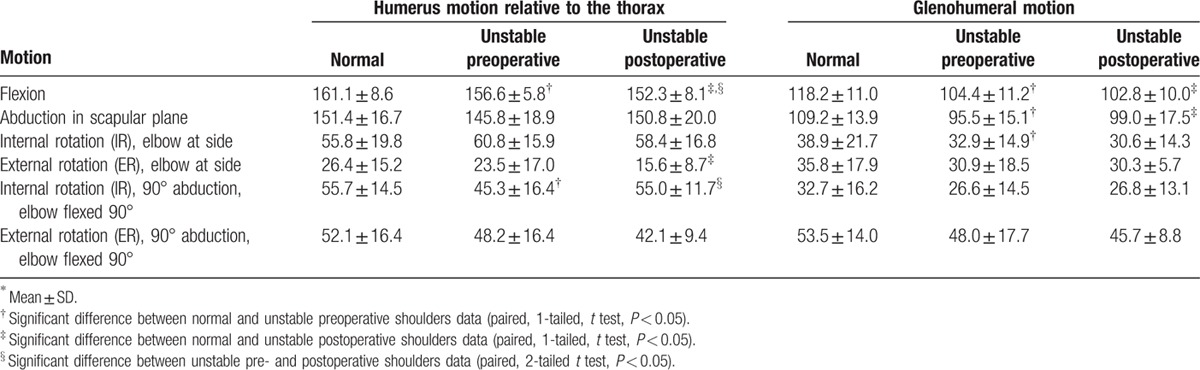
Shoulder range of motion (degree) for the normal and unstable pre- and postoperative shoulders during the 4 recorded movements (n = 33; 11 subjects, 3 trials)^∗^.

### 3.2 Glenohumeral translation

For all movements, the humeral head position of the normal and unstable pre- and postoperative shoulders was always anteriorly translated with respect to the glenoid center (Table 2). Preoperative anterior translation was higher in the unstable shoulders compared to the normal shoulders. Differences were significant for flexion and abduction movements (*P* < 0.001 for both). Anterior translation was highest during internal–external rotation, with subluxation greater than 50% on average in both pre- and postoperative ipsilateral side. Postoperatively, anterior translation for the stabilized shoulders was not significantly reduced compared to preoperative values.

**Table 2:**
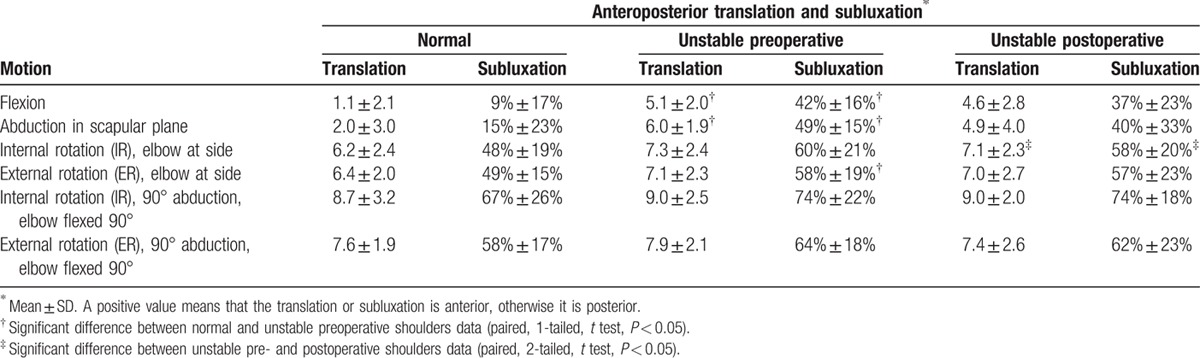
Anteroposterior translation (mm) and percentage of subluxation for the normal and unstable pre- and postoperative shoulders during the 4 recorded movements (n = 33; 1 subjects, 3 trials).

With regard to superoinferior translation, humeral head position of the normal and unstable pre- and postoperative shoulders was nearly always inferiorly translated with respect to the glenoid center. The one exception was during internal–external rotation of the arm with 90° abduction and the elbow flexed 90°, where the humeral heads of the normal and unstable postoperative shoulders were on average slightly superiorly translated. Globally, superoinferior translations remained low with a subluxation variation of ±20%. There were no significant differences between normal and unstable pre- and postoperative shoulders in this respect.

## 4 Discussion

Understanding the factors associated with postoperative stability and apprehension is important for optimizing treatment. The results of this study indicate that surgical stabilization of the shoulder lowers the risk of postoperative dislocation but leaves microinstability, confirming our hypothesis.

Preoperative ROM of the normal shoulder was higher than the unstable shoulder, probably due to pain or apprehension while executing movements. Postoperatively, motion capture analysis revealed slight mobility restriction in elevation and internal rotation with 90° abduction that could be the result of persistent either apprehension or stiffness after stabilization.

The concept of subtle preoperative glenohumeral translation has already been introduced by Patte et al^[9]^ in 1980. They defined an unstable painful shoulder as anteroinferior instability of the shoulder without any apparent history of dislocation or subluxation. The present study revealed that subtle glenohumeral translation exists postoperatively as well (in flexion [37%], abduction [40%], and external rotation with elbow at the side [58%]) even in asymptomatic patients. Such residual instability may be one explanation for residual pain, apprehension, and inability to return to sport activities following surgical stabilization.^[3,26]^

Another cause of persistent apprehension after shoulder stabilization could be central nervous system inhibition or sequelae. Previous studies have demonstrated that shoulder instability induces specific brain reorganization in functional connectivity, particularly in the primary sensorimotor cortex, dorsolateral prefrontal cortex and dorsomedial and the insula.^[6,7]^ These areas are particularly involved in complex emotional and cognitive functions including salience and anxiety,^[27]^ as well as motor resistance, and these perturbations may persist after surgery. Finally, previous research also correlated proprioceptive dysfunction to dislocation.^[8]^ During a traumatic dislocation, disruption of the shoulder capsuloligamentous complex and peripheral nerve injury can induce a decrease in kinesthetic information conveyed through proprioception, the latter playing a significant role in stabilization of a normal healthy shoulder and after any shoulder injury by helping to control muscular action.^[28]^

Given that our surgical stabilizations did not correct micromovements, other factors must be responsible for preventing postoperative dislocation. Depending on the technique, augmentation of the anteroposterior diameter of the glenoid with bone graft, the sling effect obtained by the conjoined tendon, the capsulorraphy, the repaired labrum, or remplissage could play this role.^[13]^ Interestingly, less invasive approaches have not improved results regarding stability compared to open ones; higher recurrence has been noted after arthroscopic Latarjet^[15]^ or Bankart^[29]^ procedure. Although the latter factor can be related to technical problems linked to the development of these new procedures, the bulk effect conferred by anterior scar tissue formation, already sought or reported since the beginning of shoulder stabilization,^[30]^ may also play a preponderant role. In other words, newer and mini-invasive techniques lead to less scar formation that allows more postoperative mobility but may also reduce stability proportionally.^[29]^

Finally, persistent abnormal motion between the glenoid and the humeral head as noted in the present study might be related to dislocation arthropathy which is observed with a prevalence of 36%.^[3]^ Hovelius and Saeboe^[31]^ demonstrated that development of arthritis was related to the instability phenomenon itself rather than to surgery (when properly performed). Repeated microinstability of the humeral head could lead to cartilage damage over time as the humeral head repeatedly slides against the glenoid. Conversely, some authors observed that hyperlaxity, and consequently augmented glenohumeral translation, may decrease postoperative contact pressure of the humeral head on the glenoid and thus prevent development of secondary arthritis.^[3]^

Heading toward a better understanding of the origin of instability and subsequent apprehension, postoperative management may in turn also be improved, notably in the challenging case of patients with persistent apprehension despite a clinically stable shoulder. Knowing that shoulder stabilization prevents further dislocation but does not preclude residual micromotion from occurring may avoid unnecessary physiotherapy sessions or even reoperations. Furthermore, this perspective offers a new angle/point of view of/for therapeutic approach that differs from conventional manual rehabilitation methods centered on a supposed stable shoulder. The results of this study suggest that, if persistent subtle shoulder instability is suspected, patients might actually benefit from a multidisciplinary approach including a “re-afferentation” of their shoulder based on a neuromuscular and proprioceptive work, a cognitive behavioral approach to decondition this pathological residual apprehension by making them realize residual micromotion does not necessarily lead to recurrent instability, coupled with rotator cuff reinforcement to avoid further dysfunction due to muscle fatigue, notably anterosuperior migration of the humeral head and consequent impingement.^[32]^

Cognitive behavioral therapy with gradual exposition has already demonstrated successful results in the treatment of kinesiophobia,^[33–35]^ a condition based on a reinjury fear-avoidance model initially described in low-back pain,^[36]^ further popularized in sports medicine^[37]^ and various upper limb conditions.^[38]^ Neurofeedback may be another applicable treatment modality where the patient directly visualizes his abnormal response to a negative stimulus on functional MRI or electroencephalogram, and can thereby actively correct it.^[39]^

### 4.1 Strengths and limitations

This prospective study was the first to precisely analyze pre- and postoperative micromotion in the setting of glenohumeral instability. The findings are relevant and may change the current pre-, intra-, and postoperative approach to unstable shoulders. Several surgical options were used, representing the panoply of currently available treatment options. The number of patients, due to the complexity of analysis, was adequate compared to previous shoulder instability studies.^[24,40,41]^ Moreover, patient selection was strict with exclusion of all conditions (hyperlaxity, nontraumatic onset, etc) that might affect the results.

However, they were several limitations that warrant discussion. First, the accuracy of the kinematics computation from motion capture data. Glenohumeral orientation and translation errors were respectively within 4∘ and 3 mm for each anatomical plane, which is acceptable for clinical use in the study of shoulder pathology. Although the translation error could be of significant importance for our model, we previously demonstrated that the computed translation patterns and amplitudes were in good agreement with published data.^[11,20,24]^ For comparison, Karduna et al^[42]^ reported orientation errors of 10∘ for a scapula tracker and 11.4∘ for an acromial method against bone pins. Although glenohumeral translation quantification has been studied for more than 2 decades,^[43]^ we found no other study able to report translation values at the glenohumeral joint using an external measurement system, such as motion capture. Second, the limited number of patients did not allow for comparison between the different surgical techniques. Nevertheless, the results representing the activity of a shoulder surgeon showed that all translations followed similar patterns.

## 5 Conclusion

While surgical treatment for anterior instability limits the chance of dislocation, it does not seem to restore glenohumeral translation during functional ROM. Such persistent microinstability may explain residual pain, apprehension, inability to return to activity and even emergence of dislocation arthropathy that is seen in some patients. Further research is necessary to better understand the causes, effects, and treatment of residual microinstability following surgical stabilization of the shoulder.

## References

[1] Romeo AA Cohen BS Carreira DS Traumatic anterior shoulder instability. *Orthop Clin North Am* 2001; 32:399–409. 11888135 10.1016/s0030-5898(05)70209-1

[2] Hovelius L Vikerfors O Olofsson A Bristow-Latarjet and Bankart: a comparative study of shoulder stabilization in 185 shoulders during a seventeen-year follow-up. *J Shoulder Elbow Surg* 2011; 20:1095–1101. 21602067 10.1016/j.jse.2011.02.005

[3] Lädermann A Lubbeke A Stern R Risk factors for dislocation arthropathy after Latarjet procedure: a long-term study. *Int Orthop* 2013; 37:1093–1098. 23508865 10.1007/s00264-013-1848-y PMC3664176

[4] Hovelius L Sandstrom B Saebo M One hundred eighteen Bristow-Latarjet repairs for recurrent anterior dislocation of the shoulder prospectively followed for fifteen years: study II—the evolution of dislocation arthropathy. *J Shoulder Elbow Surg* 2006; 15:279–289. 16679226 10.1016/j.jse.2005.09.014

[5] Meller R Krettek C Gosling T Recurrent shoulder instability among athletes: changes in quality of life, sports activity, and muscle function following open repair. *Knee Surg Sports Traumatol Arthrosc* 2007; 15:295–304. 16816984 10.1007/s00167-006-0114-x

[6] Cunningham G Zanchi D Emmert K Neural correlates of clinical scores in patients with anterior shoulder apprehension. *Med Sci Sports Exerc* 2015; 47:2612–2620. 26110696 10.1249/MSS.0000000000000726

[7] Haller S Cunningham G Lädermann A Shoulder apprehension impacts large-scale functional brain networks. *AJNR Am J Neuroradiol* 2014; 35:691–697. 24091445 10.3174/ajnr.A3738 PMC7965828

[8] Atef A El-Tantawy A Gad H Prevalence of associated injuries after anterior shoulder dislocation: a prospective study. *Int Orthop* 2016; 40:519–524. 26133290 10.1007/s00264-015-2862-z

[9] Patte D Bernageau J Rodineau J Unstable painful shoulders (author's transl). *Rev Chir Orthop Reparatrice Appar Mot* 1980; 66:157–165. 6450978

[10] Singer GC Kirkland PM Emery RJ Coracoid transposition for recurrent anterior instability of the shoulder. A 20-year follow-up study. *J Bone Joint Surg Br* 1995; 77:73–76. 7822401

[11] Charbonnier C Chague S Kolo FC A patient-specific measurement technique to model shoulder joint kinematics. *Orthop Traumatol Sur Res* 2014; 100:715–719. 10.1016/j.otsr.2014.06.015 25281547

[12] Coudane H Walch G Sebesta A Chronic anterior instability of the shoulder in adults. Methodology. *Rev Chir Orthop Reparatrice Appar Mot* 2000; 86 suppl 1:94–95. 11084503

[13] Young AA Maia R Berhouet J Open Latarjet procedure for management of bone loss in anterior instability of the glenohumeral joint. *J Shoulder Elbow Surg* 2011; 20 (2 suppl):S61–S69. 21145262 10.1016/j.jse.2010.07.022

[14] Bouju Y Gadea F Stanovici J Shoulder stabilization by modified Latarjet-Patte procedure: results at a minimum 10 years’ follow-up, and role in the prevention of osteoarthritis. *Orthop Traumatol Surg Res* 2014; 100 (4 suppl):S213–S218. 24703796 10.1016/j.otsr.2014.03.010

[15] Cunningham G Benchouk S Kherad O Comparison of arthroscopic and open Latarjet with a learning curve analysis. *Knee Surg Sports Traumatol Arthrosc* 2016; 24:540–545. 26658571 10.1007/s00167-015-3910-3

[16] Walch G The Walch-Duplay rating sheet for anterior instability of the shoulder. Paper presented at: SECEC/ESSSE1987; Paris.

[17] Rowe CR Patel D Southmayd WW The Bankart procedure: a long-term end-result study. *J Bone Joint Surg Am* 1978; 60:1–16. 624747

[18] Gilbart MK Gerber C Comparison of the subjective shoulder value and the Constant score. *J Shoulder Elbow Surg* 2007; 16:717–721. 18061114 10.1016/j.jse.2007.02.123

[19] Huskisson EC Measurement of pain. *J Rheumatol* 1982; 9:768–769. 6184474

[20] Charbonnier C Chague S Kolo FC Shoulder motion during tennis serve: dynamic and radiological evaluation based on motion capture and magnetic resonance imaging. *Int J Comput Assist Radiol Surg* 2015; 10:1289–1297. 25503926 10.1007/s11548-014-1135-4

[21] Grood ES Suntay WJ A joint coordinate system for the clinical description of three-dimensional motions: application to the knee. *J Biomech Eng* 1983; 105:136–144. 6865355 10.1115/1.3138397

[22] Wu G van der Helm FC Veeger HE ISB recommendation on definitions of joint coordinate systems of various joints for the reporting of human joint motion—Part II: shoulder, elbow, wrist and hand. *J Biomech* 2005; 38:981–992. 15844264 10.1016/j.jbiomech.2004.05.042

[23] Schneider P Eberly D Geometric Tools for Computer Graphics (The Morgan Kaufmann Series in Computer Graphics). San Francisco: Morgan Kaufmann; 2002.

[24] Lädermann A Chague S Kolo FC Kinematics of the shoulder joint in tennis players. *J Sci Med Sport* 2016; 19:56–63. 25481481 10.1016/j.jsams.2014.11.009

[25] Silliman JF Hawkins RJ Classification and physical diagnosis of instability of the shoulder. *Clin Orthop Relat Res* 1993; 291:7–19. 8504616

[26] Boileau P Zumstein M Balg F The unstable painful shoulder (UPS) as a cause of pain from unrecognized anteroinferior instability in the young athlete. *J Shoulder Elbow Surg* 2011; 20:98–106. 20850995 10.1016/j.jse.2010.05.020

[27] Vogt BA Pain and emotion interactions in subregions of the cingulate gyrus. *Nat Rev Neurosci* 2005; 6:533–544. 15995724 10.1038/nrn1704 PMC2659949

[28] Fyhr C Gustavsson L Wassinger C The effects of shoulder injury on kinaesthesia: a systematic review and meta-analysis. *Man Ther* 2015; 20:28–37. 25241661 10.1016/j.math.2014.08.006

[29] Chen L Xu Z Peng J Effectiveness and safety of arthroscopic versus open Bankart repair for recurrent anterior shoulder dislocation: a meta-analysis of clinical trial data. *Arch Orthop Trauma Surg* 2015; 135:529–538. 25743570 10.1007/s00402-015-2175-0

[30] Hippocrates. Works of Hippocrates with an English Translation. London: Hippocrates; 1927.

[31] Hovelius L Saeboe M Neer Award 2008: arthropathy after primary anterior shoulder dislocation—223 shoulders prospectively followed up for twenty-five years. *J Shoulder Elbow Surg* 2009; 18:339–347. 19254851 10.1016/j.jse.2008.11.004

[32] Seitz AL McClure PW Finucane S Mechanisms of rotator cuff tendinopathy: intrinsic, extrinsic, or both? *Clin Biomech* 2011; 26:1–12. 10.1016/j.clinbiomech.2010.08.001 20846766

[33] Linton SJ Andersson T Can chronic disability be prevented? A randomized trial of a cognitive-behavior intervention and two forms of information for patients with spinal pain. *Spine* 2000; 25:2825–2831.discussion 2824. 11064530 10.1097/00007632-200011010-00017

[34] Monticone M Ambrosini E Rocca B A multidisciplinary rehabilitation programme improves disability, kinesiophobia and walking ability in subjects with chronic low back pain: results of a randomised controlled pilot study. *Eur Spine J* 2014; 23:2105–2113. 25064093 10.1007/s00586-014-3478-5

[35] Morley S Eccleston C Williams A Systematic review and meta-analysis of randomized controlled trials of cognitive behaviour therapy and behaviour therapy for chronic pain in adults, excluding headache. *Pain* 1999; 80:1–13. 10204712 10.1016/s0304-3959(98)00255-3

[36] Vlaeyen JW Seelen HA Peters M Fear of movement/(re)injury and muscular reactivity in chronic low back pain patients: an experimental investigation. *Pain* 1999; 82:297–304. 10488681 10.1016/S0304-3959(99)00054-8

[37] Kvist J Ek A Sporrstedt K Fear of re-injury: a hindrance for returning to sports after anterior cruciate ligament reconstruction. *Knee Surg Sports Traumatol Arthrosc* 2005; 13:393–397. 15703963 10.1007/s00167-004-0591-8

[38] Das De S Vranceanu AM Ring DC Contribution of kinesophobia and catastrophic thinking to upper-extremity-specific disability. *J Bone Joint Surg Am* 2013; 95:76–81. 23283376 10.2106/JBJS.L.00064

[39] deCharms RC Maeda F Glover GH Control over brain activation and pain learned by using real-time functional MRI. *Proc Natl Acad Sci USA* 2005; 102:18626–18631. 16352728 10.1073/pnas.0505210102 PMC1311906

[40] Graichen H Hinterwimmer S von Eisenhart-Rothe R Effect of abducting and adducting muscle activity on glenohumeral translation, scapular kinematics and subacromial space width in vivo. *J Biomech* 2005; 38:755–760. 15713296 10.1016/j.jbiomech.2004.05.020

[41] Matsuki K Matsuki KO Yamaguchi S Dynamic in vivo glenohumeral kinematics during scapular plane abduction in healthy shoulders. *J Orthop Sports Phys Ther* 2012; 42:96–104. 22030448 10.2519/jospt.2012.3584

[42] Karduna AR McClure PW Michener LA Dynamic measurements of three-dimensional scapular kinematics: a validation study. *J Biomech Eng* 2001; 123:184–190. 11340880 10.1115/1.1351892

[43] Harryman DT II Sidles JA Clark JM Translation of the humeral head on the glenoid with passive glenohumeral motion. *J Bone Joint Surg Am* 1990; 72:1334–1343. 2229109

